# Expression of SATB1 and SATB2 in the brain of bony fishes: what fish reveal about evolution

**DOI:** 10.1007/s00429-023-02632-z

**Published:** 2023-04-01

**Authors:** Daniel Lozano, Jesús M. López, Sara Jiménez, Ruth Morona, Víctor Ruíz, Ana Martínez, Nerea Moreno

**Affiliations:** Department of Cell Biology, Faculty of Biology, University Complutense, 28040 Madrid, Spain

**Keywords:** Evolution, Fish, Sarcopterygians, Actinopterygians, Pallium, Preoptic area, Amygdala, Hypothalamus, Lungfish, Zebrafish, Cladistians, Chondrosteans, Holosteans

## Abstract

Satb1 and Satb2 belong to a family of homeodomain proteins with highly conserved functional and regulatory mechanisms and posttranslational modifications in evolution. However, although their distribution in the mouse brain has been analyzed, few data exist in other non-mammalian vertebrates. In the present study, we have analyzed in detail the sequence of SATB1 and SATB2 proteins and the immunolocalization of both, in combination with additional neuronal markers of highly conserved populations, in the brain of adult specimens of different bony fish models at key evolutionary points of vertebrate diversification, in particular including representative species of sarcopterygian and actinopterygian fishes. We observed a striking absence of both proteins in the pallial region of actinopterygians, only detected in lungfish, the only sarcopterygian fish. In the subpallium, including the amygdaloid complex, or comparable structures, we identified that the detected expressions of SATB1 and SATB2 have similar topologies in the studied models. In the caudal telencephalon, all models showed significant expression of SATB1 and SATB2 in the preoptic area, including the acroterminal domain of this region, where the cells were also dopaminergic. In the alar hypothalamus, all models showed SATB2 but not SATB1 in the subparaventricular area, whereas in the basal hypothalamus the cladistian species and the lungfish presented a SATB1 immunoreactive population in the tuberal hypothalamus, also labeled with SATB2 in the latter and colocalizing with the gen Orthopedia. In the diencephalon, all models, except the teleost fish, showed SATB1 in the prethalamus, thalamus and pretectum, whereas only lungfish showed also SATB2 in prethalamus and thalamus. At the midbrain level of actinopterygian fish, the optic tectum, the torus semicircularis and the tegmentum harbored populations of SATB1 cells, whereas lungfish housed SATB2 only in the torus and tegmentum. Similarly, the SATB1 expression in the rhombencephalic central gray and reticular formation was a common feature. The presence of SATB1 in the solitary tract nucleus is a peculiar feature only observed in non-teleost actinopterygian fishes. At these levels, none of the detected populations were catecholaminergic or serotonergic. In conclusion, the protein sequence analysis revealed a high degree of conservation of both proteins, especially in the functional domains, whereas the neuroanatomical pattern of SATB1 and SATB2 revealed significant differences between sarcopterygians and actinopterygians, and these divergences may be related to the different functional involvement of both in the acquisition of various neural phenotypes.

## Introduction

The special AT-rich sequence-binding proteins 1 and 2 (SATB1 and SATB2) belong to a family of homeodomain proteins, with a SATB domain and two CUT domains, and a homeodomain for DNA binding (Dickinson et al. 1992; Cai et al. 2003; FitzPatrick et al. 2003; Britanova et al. 2005). In particular, the CUT domain binds to specific AT-rich matrix-binding regions (Dickinson et al. 1997), which attach to the nuclear matrix, separating chromatin into topologically independent loop domains. In particular, they bind to multiple sites within chromatin loop domains and modulate their organization and structure, modifying the transcription of various genes at different levels, including in the brain (Ciejek et al. 1983; Vaughn et al. 1990; Alvarez et al. 2000; Cai et al. 2003; Dobreva et al. 2003, 2006; Britanova et al. 2005; Ahn et al. 2010). Therefore, in contrast to classical transcription factors that bind to individual target genes, SATB family members regulates target genes in a context-dependent manner, depending on its genomic occupancy and potential to form a complex with multiple partners. Moreover, this probably occurs thanks to this functional flexibility, with a highly conserved pattern in evolution.

It has been described a 61% homology between SATB1 and SATB2 at amino acid level, and in the mouse, no other homologues have been identified (Britanova et al. 2005). Firstly, it was described SATB1, regulating the expression of multiple genes in the maturating T-cells, as a global regulator of thymocyte differentiation (Dickinson et al. 1992; Yasui et al. 2002; Cai et al. 2003), but it was also detected in the fetal brain. SATB2 was described in the SATB2-associated syndrome, an autosomal dominant neurodevelopmental disorder caused by alterations in the SATB2 gene, which is a multisystem disorder characterized by significant neurodevelopmental compromise with limited to absent speech, behavioral issues, and craniofacial anomalies (reviewed in Zarate et al. 2017, 2019). In addition, its regulatory role has been shown to be crucial during early vertebrate embryogenesis (Pradhan et al. 2021). In the brain, SATB2 has been implicated in the development of different regions like the mouse neocortex (Alcamo et al. 2008; Britanova et al. 2008; Leone et al. 2015; McKenna et al. 2015), different hypothalamic subpopulations (Kurrasch et al. 2007; Blackshaw et al. 2010; Bedont et al. 2015; Ferran et al. 2015; Glendining et al. 2020; Zhang et al. 2021), and in the function of the parabrachial area (Fu et al. 2019; Jarvie et al. 2021; Karthik et al. 2022). Its expression and functional domains are highly conserved in humans, mice, chick, and zebrafish (Ahn et al. 2010; Sheehan-Rooney et al. 2010). Moreover, in the case of the SATB1 and SATB2 expression in the brain, particularly well described in mice, their expression patterns apparently do not overlap (Britanova et al. 2005).

**Fig. 1:**
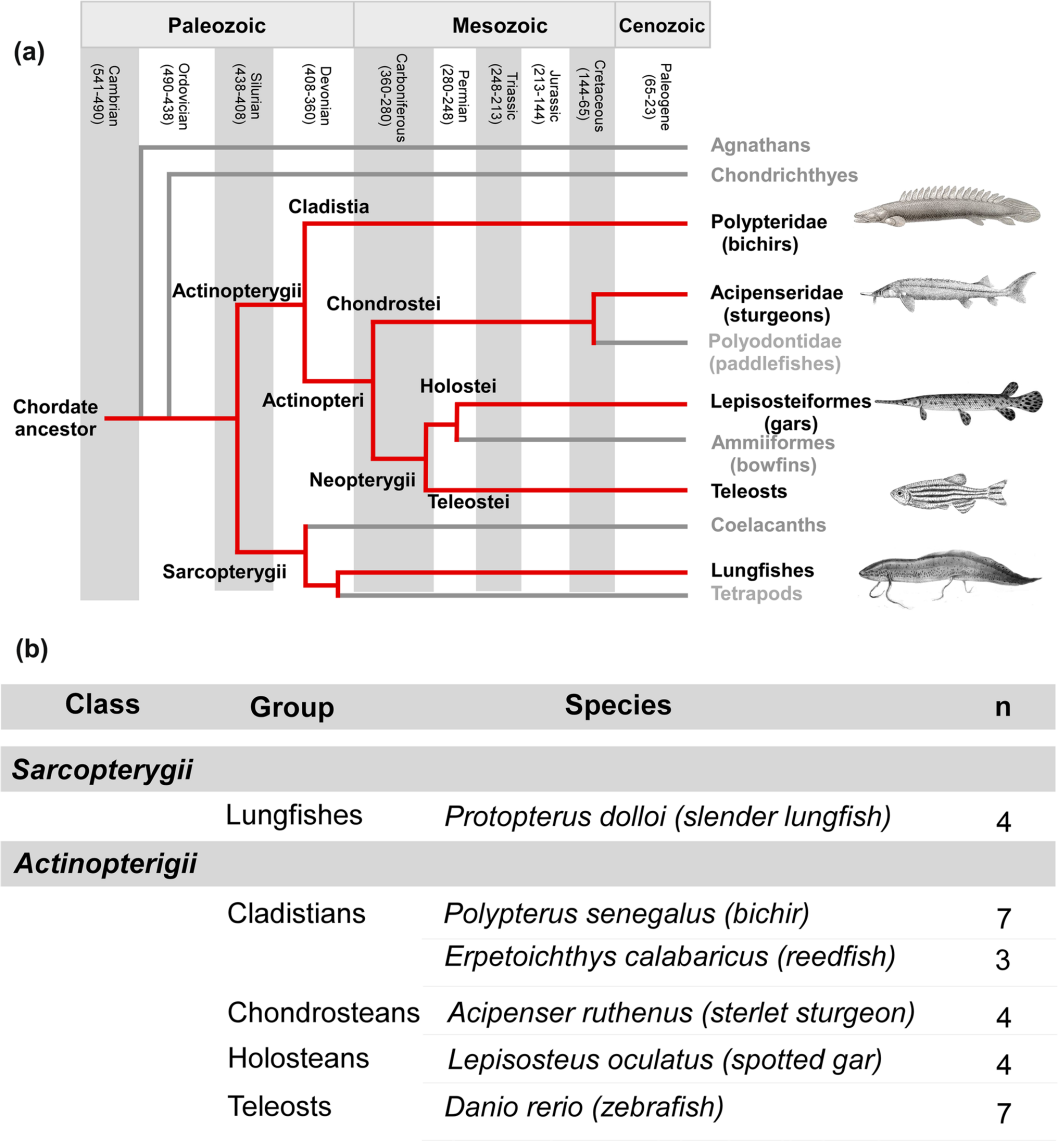
**A** Cladogram showing the phylogenetic relationships of the four major groups of vertebrates: agnathans, chondrichthyans (cartilaginous fishes), actinopterygians (ray-finned fishes), and the sarcopterygian radiation of lobe-finned fishes. **B** Number of animals (n) and species used in the present study. The fishes were purchased from the authorized commercial supplier *PezyCia* (Madrid, Spain)

Thus, considering the functional importance and versatility of these proteins from early developmental stages and in a multitude of systems including the brain, extending its knowledge to basal fish models may provide important insights. It is known that comparative expression patterns of transcription factors involved in the acquisition of the identity of specific neuronal phenotypes, or of major brain subdivisions, constitute an extremely important source of information in the analysis of brain evolution, but also improve the understanding of how the brain is organized and functions. Thus, in this context of a conserved gene family, there are currently no data on the expression pattern of SATB1 in non-mammalian models, and data on SATB2 are very scarce. In particular, there is a lack of data on the expression and function of both proteins at the base of the evolutionary scale (except SATB2 in zebrafish). Furthermore, in the case of fishes, the high diversity found implies that many of the evolutionary lineages are poorly studied anatomically. Therefore, in the present study, we have selected representative species of actinopterygian and sarcopterygian fishes that allow us to identify differences in critical evolutionary points (see Fig. 1A). On the one hand, lungfishes constitute the most basal sarcopterygian model and the only non-tetrapod anamniote sarcopterygian, therefore the closest living relatives of all tetrapods. On the other hand, during the Devonian period, basal actinopterygian fishes were divided into the classes Cladistia (polypteriform fishes) and Actinopteri, which gave rise to the subclass Neopterygii (holostean and teleost fishes), and sturgeons (Broughton et al. 2013; Betancur-R et al. 2013; Hughes et al. 2018; Du et al. 2020). Therefore, in order to infer the characteristics specific to ancestral Osteichthyes, and to the transition between actinopterygians and sarcopterygians, as well as the evolutionary adaptations of each group, the first objective of the present study was to perform an in-depth analysis of the SATB protein sequences in these evolutionarily key species. The second, and main goal was to analyze in detail their neuroanatomical distribution in the brain of the mentioned models. Additionally, we phenotypically characterized these neurons by double immunolabeling techniques with the enzyme tyrosine hydroxylase (TH), as the main catecholaminergic marker (mainly dopaminergic and noradrenergic), serotonin (5-HT), and the transcription factor Orthopedia (Otp), highly expressed in particular regions of the amygdaloid complex and the hypothalamus (Puelles and Rubenstein 2003; Domínguez et al. 2013, 2014, 2015; Moreno et al. 2012).

## Materials and methods

### Analysis of the sequence

**Fig. 2:**
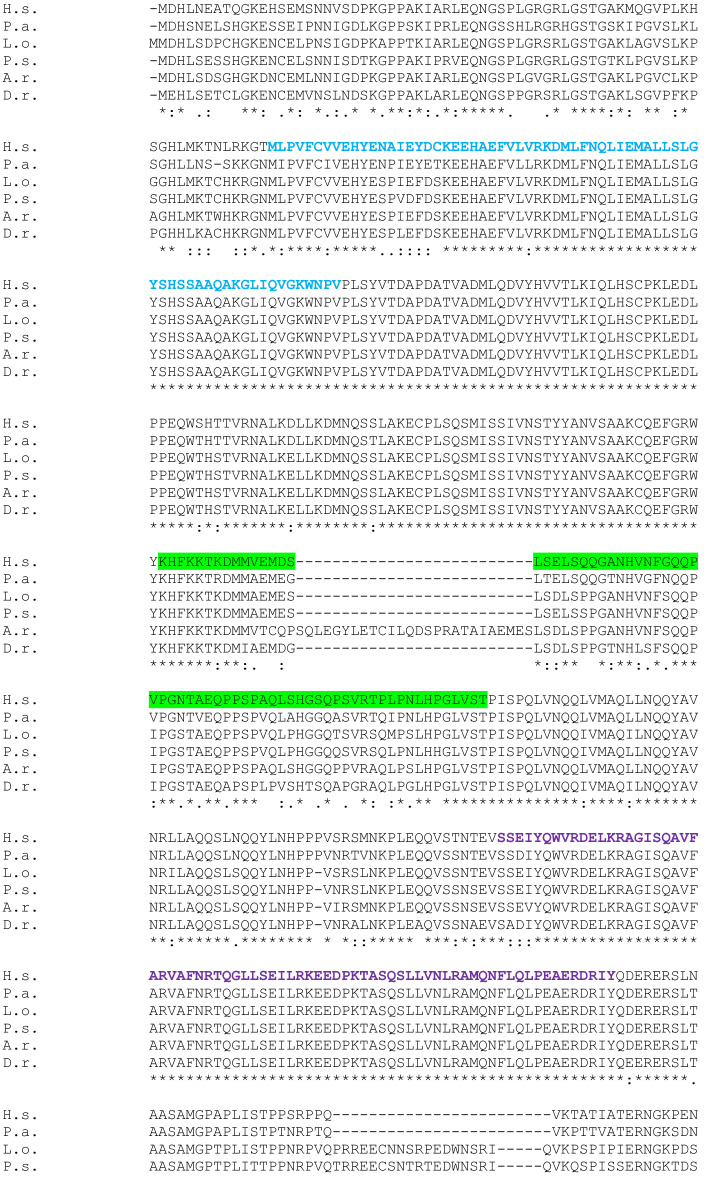

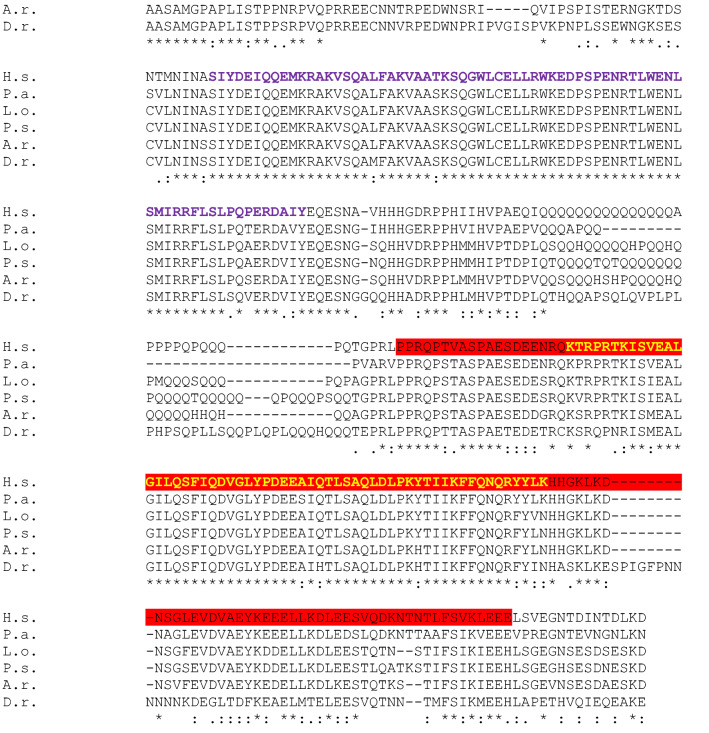
Full-length SATB1 protein sequence alignment in Homo sapiens (H.s) as a query, and Protopterus annectens (P.a), Lepisosteus oculatus (L.o), Danio rerio (D.r), Acipenser ruthenus (A.r) and Polypterus senegalus (P.s) using CLUSTALW. Identical amino acids are shown with an asterisk (*). The colon (:) and the dot (.) indicate a conserved substitution, and a semi-conserved substitution respectively. The SATB domain is shown in blue, the CUT domains are shown in purple and the homeodomain in yellow. The epitope sequence recognized by the Satb1/2 antibody (Abcam. Catalog reference ab51502) is underlined in red and the epitope sequence recognized by the Satb1 antibody (Santa Cruz Biotechnology. Catalog reference sc-376096) is underlined in green

**Fig. 3:**
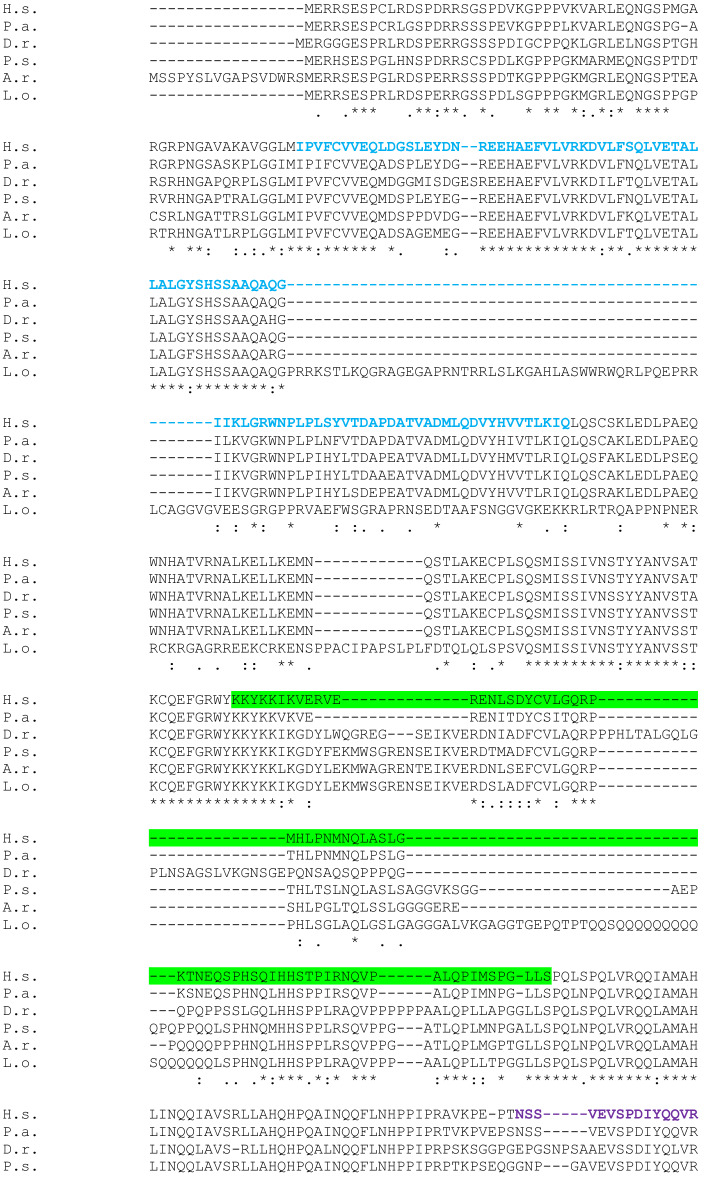

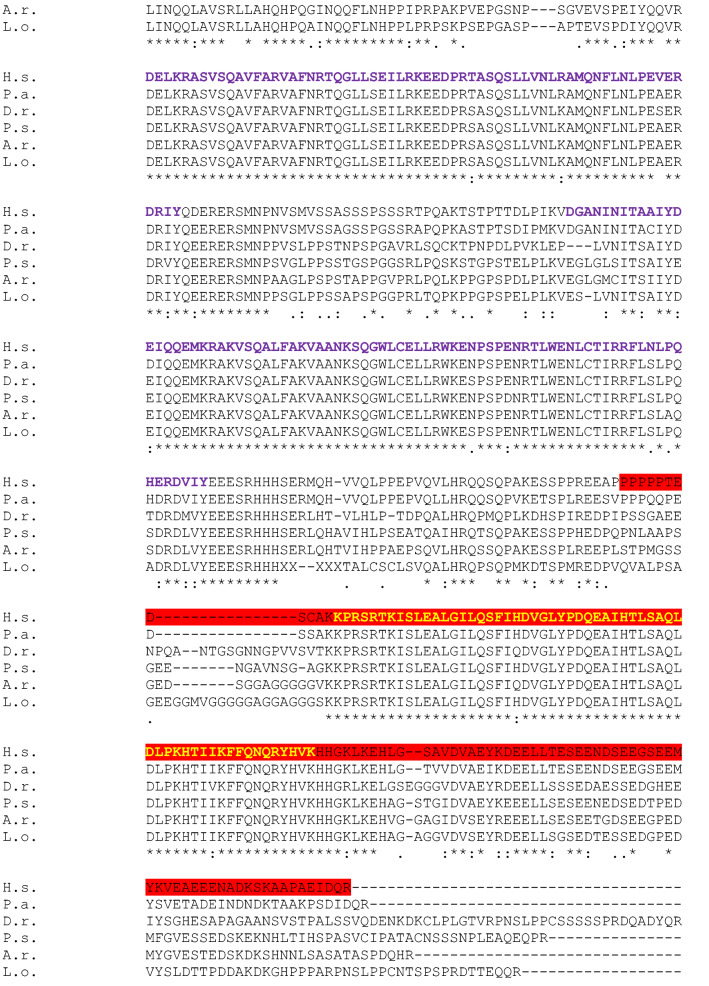
Full-length SATB2 protein sequence alignment in Homo sapiens (H.s) as a query, and Protopterus annectens (P.a), Lepisosteus oculatus (L.o), Danio rerio (D.r), Acipenser ruthenus (A.r) and Polypterus senegalus (P.s) using CLUSTALW. Identical amino acids are shown with an asterisk (*). The colon (:) and the dot (.) indicate a conserved substitution, and a semi-conserved substitution respectively. The SATB domain is shown in blue, the CUT domain is shown in purple and the homeodomain in yellow. The epitope sequence recognized by the Satb1/2 antibody (Abcam. Catalog reference ab51502) is underlined in red and the epitope sequence recognized by the Satb1 antibody (Santa Cruz Biotechnology. Catalog reference sc-376096) is underlined in green

We run a BLAST PROTEIN of the whole SATB1 and SATB2 protein sequences of *Homo sapiens* (H.s; taxid: 9606), *Protopterus annectens* (P.a; taxid: 7888), *Lepisosteus oculatus* (L.o; taxid: 7918), *Danio rerio* (D.r; taxid: 7955), *Acipenser ruthenus* (A.r; taxid: 7906) and *Polypterus senegalus* (P.s; taxid: 55291). CLUSTALW was used for multiple sequence alignments (Figs. 2 and 3). The epitope regions of the antibodies used in the present study (Table 1) were identified in the whole protein sequences of the models under analysis and BLAST PROTEIN and CLUSTALW analyses were performed for multiple sequence alignments (Table 2).

**Table 1:** List of primary antibodies, immunogens, commercial suppliers, and dilutions used in the present study

Name	Immunogen	Commercial supplier	Dilution
SATB1/2	Amino acids from 601 to the C-terminus of human SATB2 protein	Monoclonal mouse anti-Satb1/2. Abcam. Catalog reference: ab51502	1:100
SATB1	KHFKKTKDMMVEMDSLSELSQQGANHVNFGQQPVPGNTAEQPPSPAQLSHGSQPSVRTPLPNLHPGLVST	Monoclonal mouse anti-Satb1. Santa Cruz Biotechnology. Catalog reference: sc-376096	1:100
OTP	Amino acid sequence: RKALEHTVS of the C-terminal OTP protein	Polyclonal rabbit anti-Otp. Pikcell Laboratories, Kruislaan, Amsterdam, The Netherlands	1:500
TH	Protein purified from rat pheochromocytoma	Polyclonal rabbit anti-TH. Millipore. Catalog reference: AB152	1:1000
5-HT	Serotonin coupled to BSA with paraformaldehyde	Polyclonal rabbit anti-5-HT. Immunostar. Catalog reference: 20,080	1:1000

According to the species in which the primary antibody was raised, second incubations were conducted with the appropriately labeled secondary antibody: Alexa 594-conjugated goat anti-rabbit (Molecular Probes, Eugene, OR; catalog reference A-11037) and Alexa 488-conjugated goat anti-mouse (Molecular Probes; catalog reference A-21042)

**Table 2:** Pairwise sequence alignment scores and percent identity obtained in CLUSTALW and BLAST of the full-length SATB1 and SATB2 protein sequences from *Homo sapiens*, *Protopterus annectens*, *Acipenser ruthenus*, *Lepisosteus oculatus*, *Danio rerio* and *Polypterus senegalus* using as queries Satb1/2 epitope antibody sequence (Abcam. Catalog reference: ab51502) and Satb1 epitope antibody sequence (Santa Cruz Biotechnology. Catalog reference: sc-376096)

Sequences alignment	SATB1/2 EPITOPE ANTIBODY SEQUENCE		SATB1 EPITOPE ANTIBODY SEQUENCE	
	Alignment score in CLUSTALW	Percent identity in BLAST	Alignment score in CLUSTALW	Percent identity in BLAST
SATB1				
vs *Homo sapiens*	56.0606	63.71%	100	100%
vs *Protopterus annectens*	56.0606	64.35%	78.5714	78.57%
vs *Acipenser ruthenus*	54.5455	62.28%	78.5714	60.42%
vs *Lepisosteus oculatus*	56.8182	64.91%	77.1429	77.14%
vs *Danio rerio*	47.7273	54.47%	67.1429	67.14%
vs *Polypterus senegalus*	55.303	64.04%	80	80%
SATB2				
vs *Homo sapiens*	100	100%	27.1429	36.84%
vs *Protopterus annectens*	84.0909	85.07%	22.8571	39.13%
vs *Acipenser ruthenus*	67.4242	82.46%	22.8571	NSSF
vs *Lepisosteus oculatus*	62.8788	75%	20	NSSF
vs *Danio rerio*	60.6061	71.77%	20	NSSF
vs *Polypterus senegalus*	66.6667	76.47%	24.2857	NSSF

*NSSF* no significant similarity found

### Tissue handling

Representative species of the main sarcopterygian and actinopterygian fish were used (see Fig. 1). The animals were obtained from licensed suppliers (PezyCia, Madrid, Spain). Information about the number of the specimens used in the present study is shown in Fig. 1B. The animals were deeply anesthetized with 0.1% tricaine methanesulfonate solution (MS222, pH 7.4; Sigma-Aldrich, Steinheim, Germany) by immersion.

The brains and rostral spinal cords were removed after fixation by transcardial perfusion of the animals with 4% paraformaldehyde in a 0.1 M phosphate buffer (PB, pH 7.4) and subsequently cryoprotected in a solution of 30% sucrose in PB for 4–6 h at 4 ºC. The brains and spinal cords were cut at 30–40 μm thickness, in transverse or sagittal planes, on a freezing microtome, after embedded in a solution of 20% gelatin with 30% sucrose in PB.

### Immunofluorescence analysis

Single and combined immunofluorescence for detection of SATB1 or SATB1/2 and OTP, TH, or 5-HT was carried out. Information about the commercial specifications, immunogen information, the dilution used, and the secondary antibodies is detailed in Table 1. In all cases, the antibodies were diluted in PB containing 0.5% Triton X-100 and the sections were mounted with fluorescence mounting medium (Santa Cruz; SC-24941 or Vectashield H-1500). All antibodies were tested by omission or by incubation with preimmune mouse or rabbit sera instead of the primary antibody and no residual staining was observed.

### Imaging

The sections were analyzed with the microscopes: Olympus BX51, Olympus FV 1200 and Leica sp-2 AOBS confocal. The figure preparation was done with Adobe Photoshop CS6 (Adobe Systems, San Jose, CA) and Canvas X (ACD Systems, Canada).

## Results

### Sequence analysis

The models analyzed by NCBI BLAST PROTEIN and CLUSTALW were *Homo sapiens* (H.s; taxid: 9606) as a query, and *Protopterus annectens* (P.a; taxid: 7888), *Lepisosteus oculatus* (L.o; taxid: 7918), *Danio rerio* (D.r; taxid: 7955), *Acipenser ruthenus* (A. r; 7906) and *Polypterus senegalus* (P.s; taxid: 55291). The analysis was carried out based on the whole sequence proteins that were aligned using CLUSTALW to establish comparisons of the SATB1 (Fig. 2) and SATB2 (Fig. 3) proteins among these species. In addition, the epitope regions of the antibodies used in the expression patterns description were identified in the whole protein sequences of the models under analysis (see color code in Figs. 2 and 3).

The SATB1 sequence alignment in BLAST indicated that the lungfish *P. annectens* shares 83,63% sequence identity with human SATB1*,* the holostean *L. oculatus* 81,10%, the teleostean *D. rerio* 72,48%, the sturgeon *A. ruthenus* 79,08%, and the polypteriform *P. senegalus* 81,16%. In all cases, the sequences analyzed showed a SATB domain, two CUT domains and a homeobox domain (see color code in Fig. 2). In particular, the conservation of these domains is especially high in all models.

The SATB2 sequence alignment in BLAST indicated that the lungfish *P. annectens* share 88,18% sequence identity with human SATB2*,* the holostean *L. oculatus* 64,97%, the teleostean *D. rerio* 68,25%, the sturgeon *A. ruthenus* 75,49%, and the polypteriform *P. senegalus* 75,16%. In all cases, the sequences analyzed also showed a SATB domain, two CUT domains and a homeobox domain (see color code in Fig. 3). Comparatively, the SATB2 sequence showed a lower degree of conservation than SATB1 with respect to the human sequence in all models analyzed.

The subsequent analysis to ensure the reliability of the expression analysis performed was to examine the epitopes of the SATB1 and SATB2 protein sequences, from which the antibodies used in the present study were generated (see Tables 1 and 2).

In the case of the antibody developed against SATB1/2 (monoclonal mouse anti-Satb1/2. Abcam. Catalog reference: ab51502), when we compared this epitope sequence with the complete SATB1 sequence (see Table 2), we observed that the alignment score is around 50 (including human, dropping to 47 in zebrafish), and the percent identity is around 60%. The alignment to the SATB2 sequence revealed that this antibody is recognizing SATB2 in all fish models (see Table 2), being especially striking the case of lungfish, where the score rises to 84 and the percent identity to 85%, which confirms the high degree of conservation of the sequence. Therefore, it cannot be excluded that this antibody can recognize both proteins, although in the case of SATB1 with much lower affinity, attending to the sequence.

The comparison of the sequence of the epitope developed specifically against SATB1 (monoclonal mouse anti-Satb1. Santa Cruz Biotechnology; Catalog reference: sc-376096) revealed that this antibody is recognizing SATB1 in all models (the score is around 80 in all cases, being the lowest in zebrafish with 67; see Table 2). The comparison of this epitope with SATB2 showed that the alignment is around 20 in all cases (including humans, which drops to 27 and the percent identity to 36.8%), and the percent of identity in actinopterygians results in no significant similarity. This strongly supports the specificity of this antibody in the exclusive identification of SATB1 protein.

Although the analyses have demonstrated that both proteins SATB1 and SATB2 are present in the teleost *D. rerio*, the antibody against the former protein yielded no results in the immunofluorescence experiments. Considering that the gene *Satb1* is present in *D. rerio*, annotated in the forms *a* and *b* (ZFIN:ZDB-GENE-031010-36 and ZFIN:ZDB-GENE-030131-7105), and that the antibody against SATB1 used showed a high degree of similarity in the epitope sequence (see Table 2 and Fig. 2), we believe this discrepancy could be due to an effect of protein folding in this teleost model, as opposed to the rest of the models analyzed. Therefore, these data have not been included in Table 3.

**Table 3:** Summary of the distribution of Satb1 and Satb1/2 labeling in different areas of the central nervous system of the main groups of fishes studied

	Cladistians		Chondrosteans		Holosteans		Teleosts	Lungfishes	
	Satb1	Satb1/2	Satb1	Satb1/2	Satb1	Satb1/2	Satb1/2	Satb1	Satb1/2
FOREBRAIN									
Olfactory bulb	** + **	**−**	** + **	**−**	** + **	**−**	**−**	**−**	**−**
Pallium	**−**	**−**	**−**	**−**	**−**	**−**	**−**	** + **	** + **
Subpallial area Vd/Striatum	** + **	** + **	** + **	** + **	** + **	**−**	** + **	**−**	**−**
Subpallial area Vv/Septum	**−**	** + **	** + **	** + **	** + **	** + **	** + **	** + **	** + **
Subpallial area Vs-Vp/Amygdaloid complex	** + **	** + **	** + **	** + **	** + **	** + **	** + **	** + **	** + **
Preoptic area	** + **	** + **	** + **	** + **	** + **	** + **	** + **	** + **	** + **
Paraventricular region	** + **	**−**	**−**	**−**	**−**	**−**	**−**	**−**	**−**
Subparaventricular region	**−**	** + **	**−**	** + **	**−**	** + **	** + **	**−**	** + **
Tuberal hypothalamus	** + **	**−**	**−**	**−**	**−**	**−**	**−**	** + **	** + **
Mamillary hypothalamus	** + **	**−**	** + **	**−**	**−**	**−**	**−**	**−**	**−**
Prethalamus	** + **	**−**	** + **	**−**	** + **	**−**	**−**	** + **	** + **
Posterior tubercle	**−**	**−**	** + **	**−**	**−**	**−**	**−**	**−**	** + **
Thalamus	** + **	**−**	** + **	**−**	** + **	**−**	**−**	** + **	** + **
Pretectum	** + **	**−**	** + **	**−**	** + **	**−**	**−**	** + **	**−**
BRAINSTEM									
Optic Tectum	** + **	**−**	** + **	**−**	** + **	**−**	** + **	**−**	**−**
Torus semicircularis	** + **	**−**	** + **	**−**	** + **	**−**	**−**	** + **	** + **
Mesencephalic tegmentum	** + **	**−**	** + **	**−**	** + **	**−**	** + **	** + **	** + **
Cerebellum	**−**	**−**	**−**	**−**	**−**	**−**	**−**	**−**	**−**
Central gray	** + **	**−**	** + **	**−**	** + **	**−**	** + **	** + **	** + **
Superior raphe nucleus/Interpeduncular nucleus	**−**	** + **	**−**	**−**	**−**	**−**	**−**	**−**	**−**
Reticular formation	** + **	**−**	** + **	**−**	**−**	** + **	**−**	** + **	** + **
Octaval area	**−**	**−**	**−**	**−**	**−**	** + **	**−**	**−**	**−**
Solitary tract nucleus	** + **	**−**	** + **	**−**	** + **	**−**	**−**	**−**	**−**
SPINAL CORD	**−**	**−**	**−**	**−**	**−**	**−**	**−**	**−**	**−**

( +) Presence of labeling; (–) absence of labeling

### Expression pattern

**Fig. 4:**
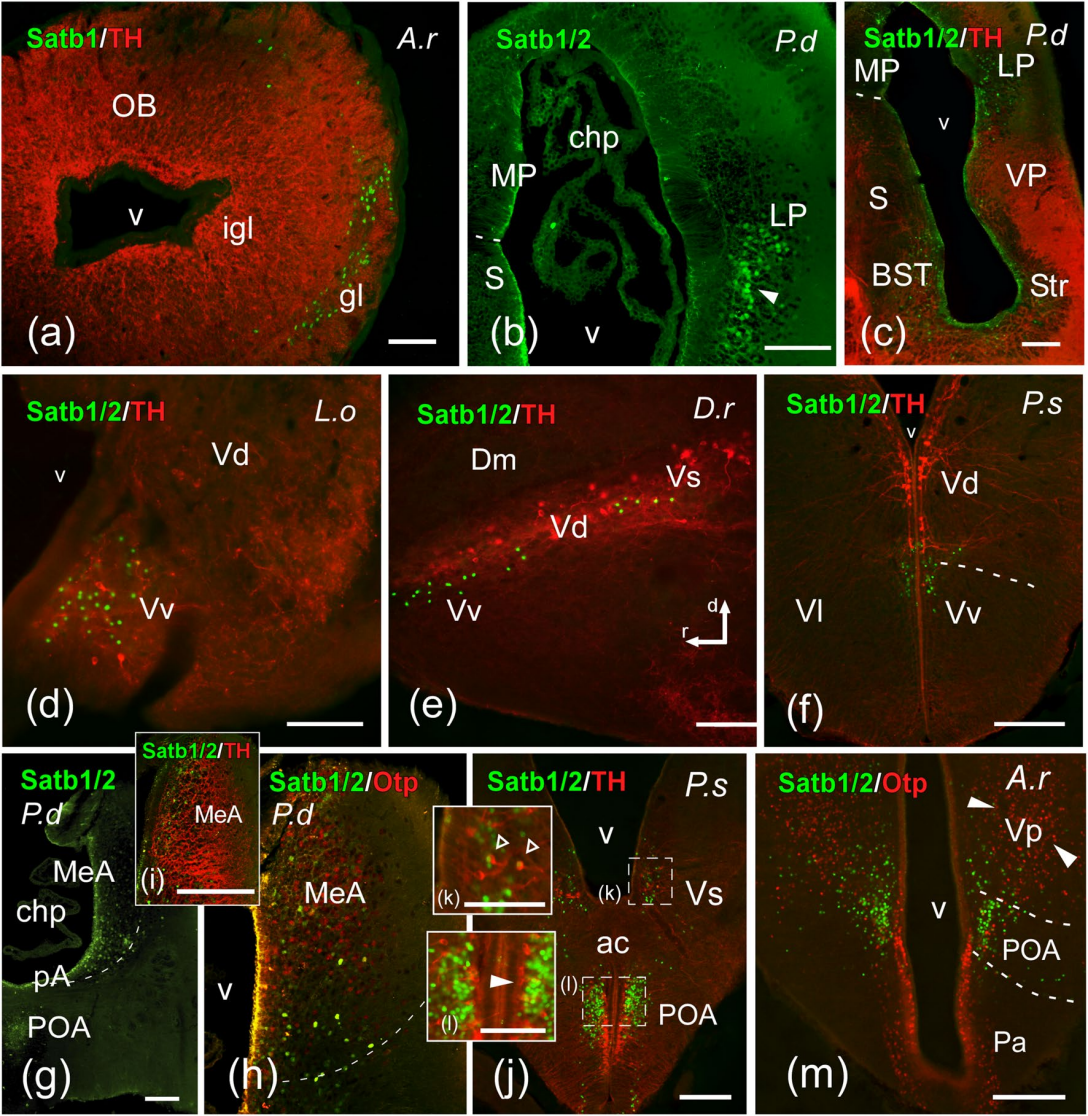
Photomicrographs of transverse (**a**–**d**, **f**–**m**) or sagittal (**e**) double-labeled sections through telencephalic areas of *Protopterus dolloi* (P.d), *Danio rerio* (D.r), *Lepisosteus oculatus* (L.o), *Polypterus senegalus* (P.s), and *Acipenser ruthenus* (A.r), showing the location of SATB1/2 (green color) alone or in combination with Otp or TH (red color) immunoreactivity. The markers and the species are indicated in each image. In the sagittal section the rostrocaudal and dorsoventral axis are indicated by arrows. **a** SATB1-ir cells in the glomerular layer of the olfactory bulb of *A. ruthenus*. **b**, **c** SATB1/2-ir cells in the pallium and subpallium of *P. dolloi*. **d**–**f** Subpallial derivatives of actinopterygian fishes labeled for SATB1/2. **g**–**i** The medial amygdala in *P. dolloi*, labeled for SATB1/2. **j**–**m** Caudal subpallial areas and the preoptic area, showing SATB1/2 immunoreactivity; empty arrowheads in (**k**) point to double labeled cells, white arrowheads in (**l**) and (**m**) point to TH-ir cells in POA and Otp-ir cells in Vp respectively. For abbreviations, see list. Scale bar: **a**, **b**, **e**, **f** = 200 µm; **c**, **d**, **g** = 100 µm

**Fig. 5:**
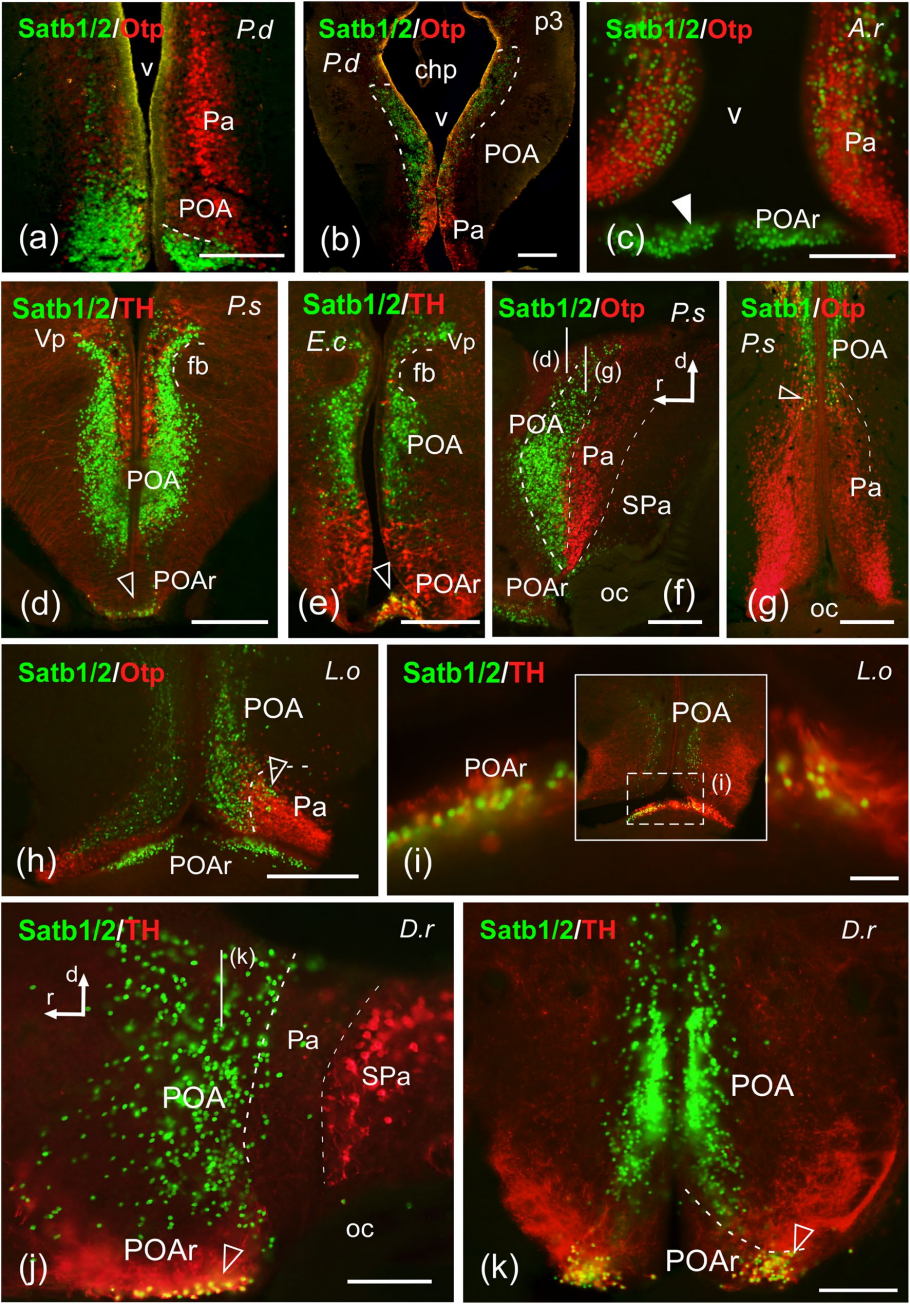
Photomicrographs of transverse (**a**–**e**, **f**–**i**, **k**) and sagittal (**f**, **j**) double-labeled sections of the preoptic area of *Protopterus dolloi* (P.d), *Erpetoichthys calabaricus* (E.c), *Danio rerio* (D.r), *Lepisosteus oculatus* (L.o), and *Polypterus senegalus* (P.s) showing the location of SATB1/2 or SATB1 (green color) alone or in combination with Otp or TH (red color) immunoreactivity. The markers and the species are indicated in each image. In the sagittal sections the rostrocaudal and dorsoventral axis are indicated by arrows. **a**, **b** SATB1/2 labeling in the POA of lungfish. **c** SATB1/2-ir cells in the rostral portion of POA of *A. ruthenus*; white arrowhead points to SATB1/2-ir cells in POAr. **d**–**f** SATB1/2 immunoreactivity in the POA of cladistians; empty arrowheads in (**d**) and (**e**) point to cells also labeled for TH. **g** SATB1-ir cells in the POA of *P. senegalus*; empty arrowhead points to double labeled cells. **h**, **i** SATB1/2-ir cells in the POA of *L. oculatus*; empty arrowhead in (**h**) and (**i**) points to double labeled cells. **j**, **k** SATB1/2-labeled cells in the POA of D. rerio; the line in (**j**) marks the level of (**k**), empty arrowheads point to double labeled cells. For abbreviations, see list. Scale bars: = 200 µm

**Fig. 6:**
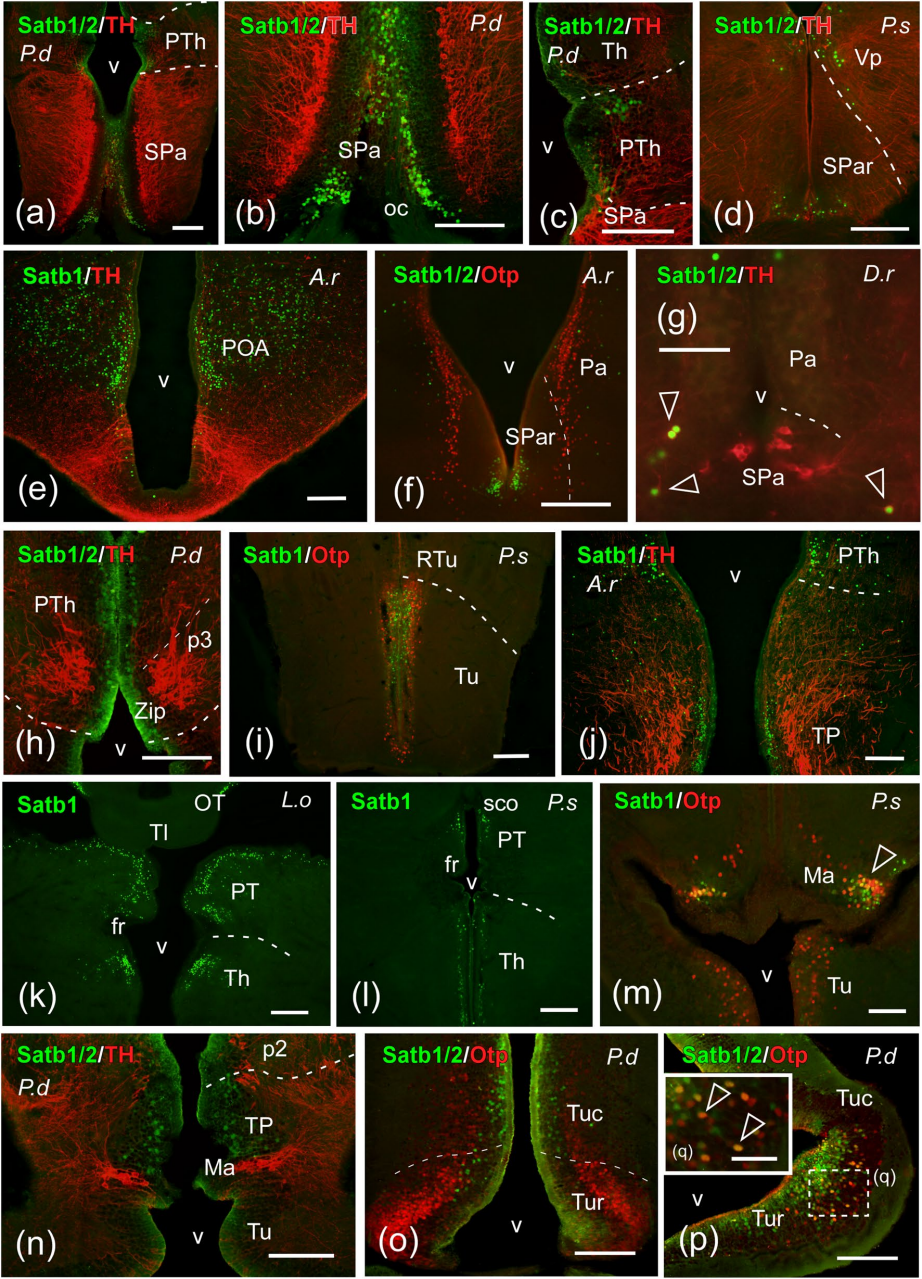
Photomicrographs of transverse double-labeled sections through hypothalamic and diencephalic areas of *Protopterus dolloi* (P.d), *Polypterus senegalus* (P.s), *Acipenser ruthenus* (A.r), *Danio rerio* (D.r), and *Lepisosteus oculatus* (L.o) showing the location of SATB1/2 or SATB1 (green color) alone or in combination with Otp or TH (red color) immunoreactivity. The markers and the species are indicated in each image. **a**–**c** SATB1/2 labeling in cells of the subparaventricular hypothalamic region (**a**, **b**) and the prethalamic and thalamic areas (**c**) of *P. dolloi*. **d** SATB1/2 cells in the rostral part of the subparaventricular region of *P. senegalus*. **e**, **f** Immunoreactive cell nuclei in the preoptic area and the subparaventricular region of *A. ruthenus*. **g** SATB1/2-ir cells in the subparaventricular region of *D. rerio*; empty arrowheads point to double labeled cells. **h** Labeled cells in the prethalamus within the alar prosomere 3 of the lungfish. **i** Tuberal hypothalamic region of *P. senegalus* showing SATB1-ir cells. **j** SATB1 labeling in the prethalamus and posterior tubercle of the sturgeon. **k**, **l** SATB1 immunoreactivity in cells of the thalamus and pretectum of *L. oculatus* (**k**) and *P. senegalus* (**l**). **m** SATB1-ir cells in the mamillary region of *P. senegalus*; empty arrowhead points to double labeled cells. **n**–**q** Labeled cells in the posterior tubercle (**n**) and the tuberal hypothalamic region (**o**–**q**) of the lungfish; empty arrowheads in **q** point to double labeled cells. For abbreviations, see list. Scale bar: **a**–**k**, **n**–**t** = 200 µm; **m**, **l** = 100 µm; **u** = 50 µm

**Fig. 7:**
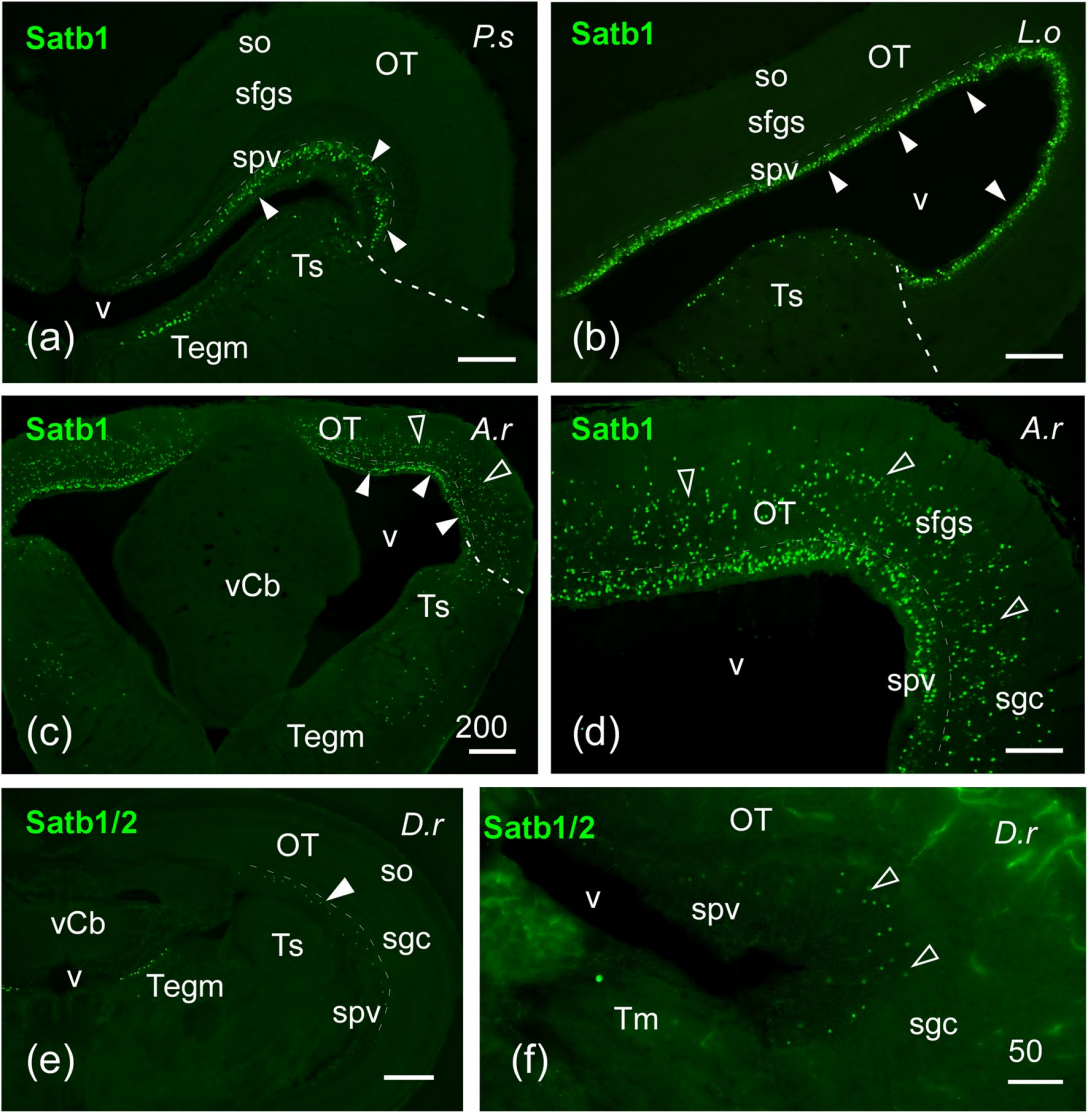
Photomicrographs of transverse single-labeled sections through mesencephalic areas of *Polypterus senegalus* (P.s), *Lepisosteus oculatus* (L.o), *Acipenser ruthenus* (A.r), and *Danio rerio* (D.r) showing the location of SATB1/2 or SATB1 immunoreactivity. The markers and the species are indicated in each image. **a**–**f** SATB1-ir cells in the periventricular layer of the optic tectum (spv), torus semicircularis and tegmentum of *P. senegalus* (**a**), *L. oculatus* (**b**), and *A. ruthenus* (**c**, **d**), the latter also with labeled cells in more superficial layers of the optic tectum; white arrowheads point to cells in spv, empty arrowheads in (**c**) and (**d**) point to cells in other tectal layers. **e**, **f** SATB1/2-ir cells in the optic tectum and tegmentum of *D. rerio*; arrowheads point to periventricular cells. For abbreviations, see list. Scale bars = 200 µm (**c**), 100 µm (**a**, **b**, **d**, **e**), 50 µm (**f**)

**Fig. 8:**
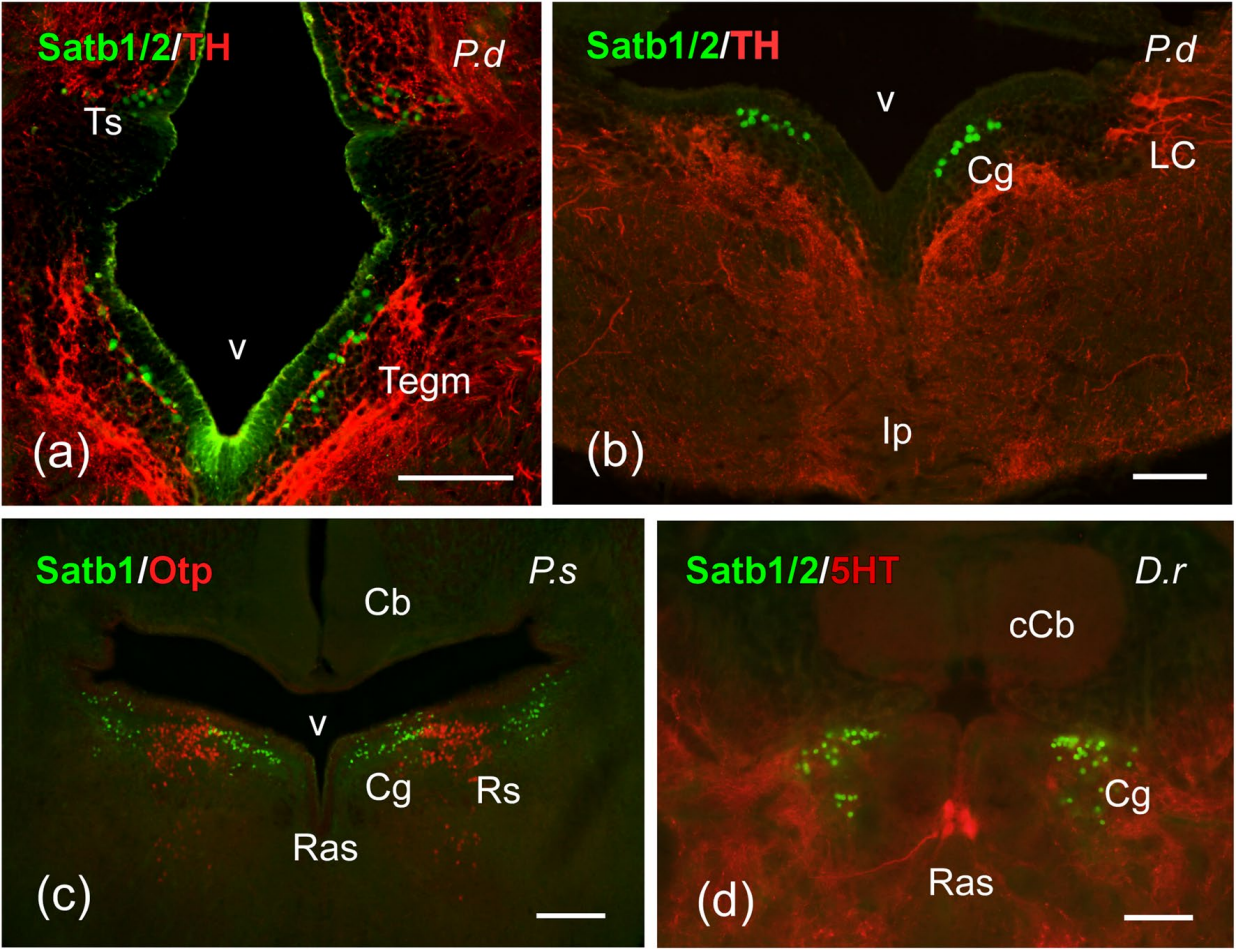
Photomicrographs of transverse single- and double-labeled sections through mesencephalic and rhombencephalic areas of *Protopterus dolloi* (P.d), *Polypterus senegalus* (P.s), and *Danio rerio* (D.r) showing the location of SATB1/2 or SATB1 (green color) alone or in combination with TH, Otp or 5-HT (red color) immunoreactivity. The markers and the species are indicated in each image. **a**, **b** SATB1/2-ir cells in the torus semicircularis and mesencephalic tegmentum (**a**), and in the central gray (**b**) of *P. dolloi*. **c** Populations of SATB1-ir cells in the central gray and superior reticular nucleus of *P. senegalus*. **d** SATB1/2-labeled cells in the central gray of *D. rerio*. For abbreviations, see list. Scale bars = 200 µm (**c**), 100 µm (**a**, **b**, **d**, **e**), 50 µm (**f**)

**Fig. 9:**
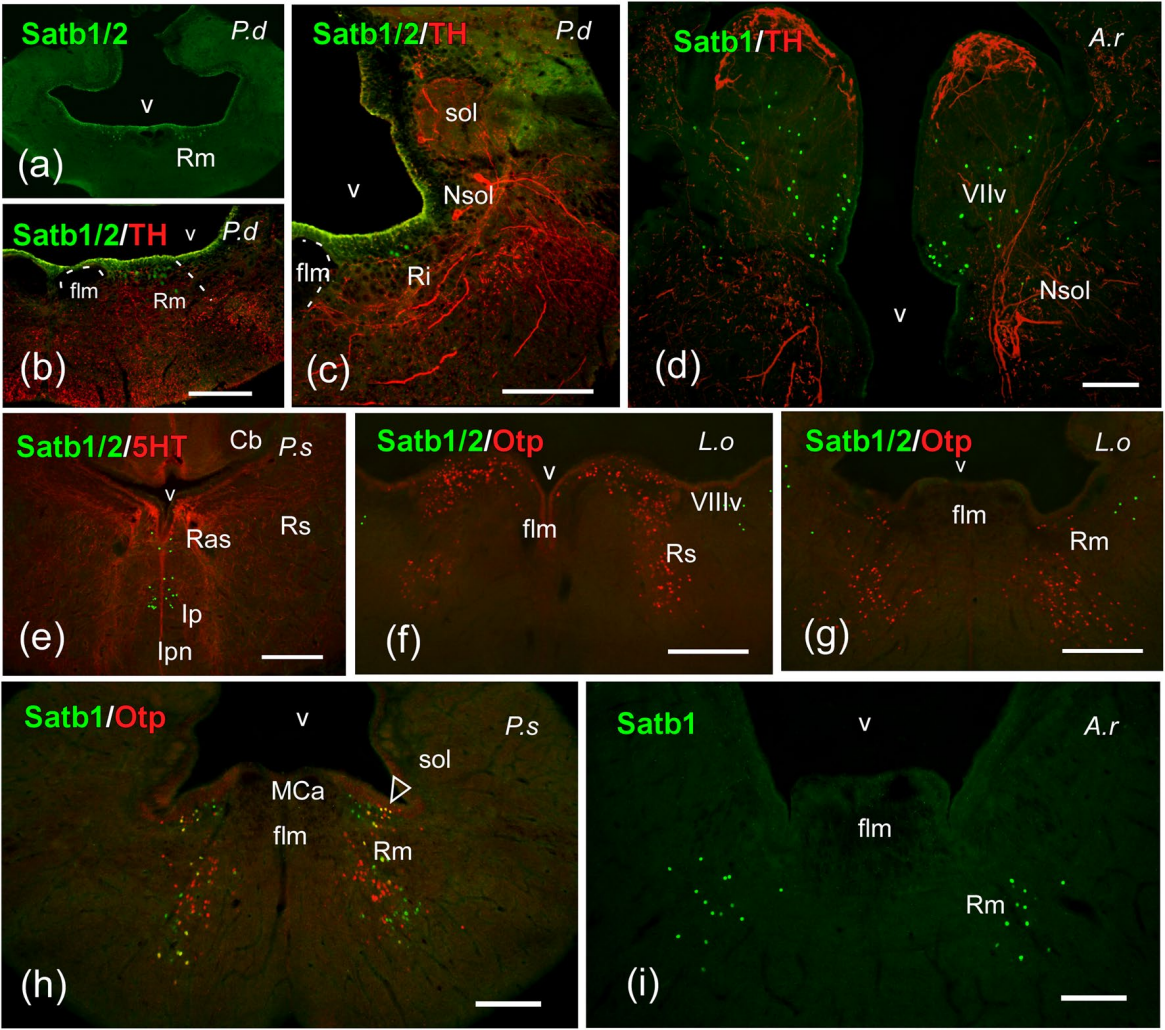
Photomicrographs of transverse single- and double-labeled sections through rhombencephalic areas of *Protopterus dolloi* (P.d), *Acipenser ruthenus* (A.r), *Polypterus senegalus* (P.s), and *Lepisosteus oculatus* (L.o) showing the location of SATB1/2 or SATB1 (green color) alone or in combination with TH, 5-HT or Otp (red color) immunoreactivity. The markers and the species are indicated in each image. **a**–**c** SATB1/2-ir cells in the reticular formation of *P. dolloi*. **d** Population of SATB1-ir cells in the facial lobe and solitary tract nucleus of *A. ruthenus*. **e** SATB1/2-ir cells in the interpeduncular nucleus of *P. senegalus*. **f**, **g** Populations of SATB1/2-ir cells in the ventral octavolateral area (**f**) and the median reticular nucleus (**g**) of *L. oculatus*. **h**, **i** Reticular cells labeled with SATB1 in *P. senegalus* (**h**) and *A. ruthenus* (**i**); arrowheads in (**h**) point to double labeled cells. For abbreviations, see list. Scale bars = 200 µm (**c**), 100 µm (**a**, **b**, **d**, **e**), 50 µm (**f**)

**Fig. 10:**
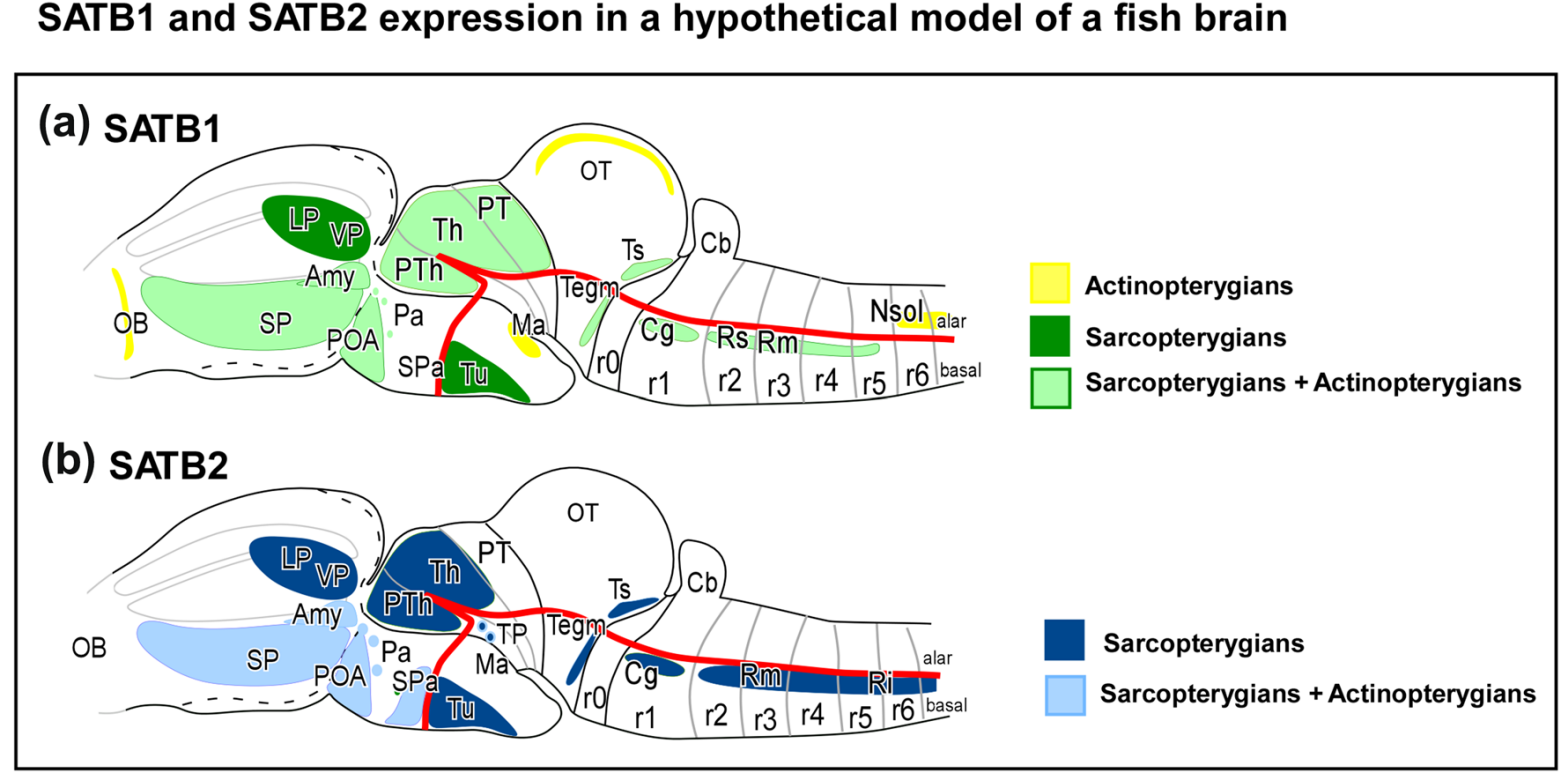
Schematic representation of SATB1 (**A**) and SATB2 (**B**) expression patterns in a model vertebrate. The schemes represent the main regions of expression found in the present study. The particular exceptions have not been included for simplicity but are detailed in the text and in Table 3. The correspondence of the color code is indicated on the right. The anatomical representation does not correspond to any particular model. See list for abbreviations

The neuroanatomical nomenclature used in the present work is similar to previous studies with the same sarcopterygian and actinopterygian fish species (López et al. 2019, 2022; Lozano et al. 2019; Jiménez et al. 2020). The distribution of the immunoreactive labeling for SATB1, SATB1/2, OTP, TH and 5-HT (Figs. 4, 5, 6, 7, 8, 9) was analyzed in relation to the main prosomeric brain regions (Puelles and Rubenstein 2015), adapted for each model. In addition, the SATB1 and SATB2 expression patterns are represented in sagittal drawings, with special reference to the neuromeric (segmental) regionalization of the brain (Fig. 10) and summarized in Table 3.

### Distribution of SATB1 and SATB2

*Telencephalon*: SATB1/2 expression was analyzed in the telencephalon in combination with markers such as TH and OTP (Figs. 4 and 5). The olfactory bulbs showed only SATB1-immunoreactive (SATB1-ir) cell nuclei in the glomerular layer of cladistians species, *A. ruthenus* and *L. oculatus*, in a lateral position (Fig. 4a).

In the telencephalon of the lungfish *Protopterus dolloi*, labeling of SATB1 and SATB2 was observed in the ventral and lateral regions of the pallium (in Fig. 4b, c), while the actinopterygian species studied lacked any pallial labeling. In the subpallium, the lateral septal area and the bed nucleus of stria terminalis in lungfish (Fig. 4c) and its homolog in ray-finned fishes—the ventral part of the ventral telencephalic area (Vv)—showed cell nuclei labeled with both antibodies in all fish species studied, with the exception of cladistians for SATB1 (Fig. 4d–f). In addition to this, codistributing with TH-ir cells, the caudal levels of the dorsal part of the ventral telencephalic area (Vd) housed scattered cells labeled with both antibodies in all actinopterygian fishes studied, with the exception of *L. oculatus*, whose Vd showed no SATB2 cells (Fig. 4d–f). In contrast, its homolog located in the striatum of lungfish was devoid of any labeling. At caudal telencephalic levels, scattered SATB1- and SATB2-ir cells were detected in the region described as medial amygdala in lungfish (Fig. 4g–i), and in its homolog in actinopterygian fishes, the supracommissural and posterior nuclei of the ventral telencephalic area (Vs-Vp) (Fig. 4j, k, m), a region that was clearly identified in combination with TH (Fig. 4i–k) and OTP (Fig. 4h, m). In particular, certain degree of colocalization of SATB2 and TH was detected in this region in cladistians (see empty arrowheads in Fig. 4k).

The preoptic area of all species of bony fish studied showed a prominent SATB1/2 labeling (Figs. 4j–m, 5 and 6e). Two subpopulations can be observed in this region: a caudal one (following the prosomeric view; dorsal in classical view), more numerous and densely packed, and without colocalization with the TH-ir cells of this region, and a rostral one (POAr; ventral in classical view), in the acroterminal domain, many of whose cells were doubly labeled with TH (Fig. 5d, e, h–k). Moreover, the combination with TH and OTP revealed the boundaries with the paraventricular domain of the alar hypothalamus (Fig. 5a–c, f, g).

*Hypothalamus and diencephalon*: In the alar hypothalamus, chondrostean (Fig. 5c) and cladistian species presented some scattered SATB1-ir cells in the dorsal paraventricular region, and in this former, some of them were doubly labeled with OTP (see empty arrowhead in Fig. 5g). In the subparaventricular region, by contrast, SATB1/2-ir cells were observed in the rostral part of the suprachiasmatic nucleus of all fish species studied (Fig. 6a, b, d, f), some of which were doubly labeled with the TH antibody in *D. rerio* (Fig. 6g). Regarding the basal hypothalamus, the cladistian species housed a population of SATB1-labeled cells in the tuberal region (Fig. 6i), while in the case of the lungfish this hypothalamic region showed SATB1/2-ir cells (Fig. 6o), some of them colocalizing with OTP in the rostral portion, but only at caudal levels (Fig. 6p, q). In addition, SATB1-ir cells were observed in the mamillary region of the chondrostean *A. ruthenus* and the cladistian species exclusively, the latter possessing certain degree of colocalization with OTP (Fig. 6m).

In the diencephalon, SATB1-ir labeling was detected in cell nuclei of the alar prethalamic, thalamic, and pretectal areas of all fish species studied (excluding *D. rerio*) (Fig. 6j–l), being the lungfish the only species that also showed SATB2 labeling in the prethalamus and the thalamus (Fig. 6c, h). In addition to these alar diencephalic domains, the basal posterior tubercle of the chondrostean *A. ruthenus* presented a periventricular population of SATB1-ir cells (Fig. 6j), while in the case of the lungfish the immunoreactive cell nuclei of this region were labeled with SATB1/2 (Fig. 6n).

*Brainstem*: Within the alar midbrain, many SATB1-ir cells were observed in the tectal periventricular layer of cladistian species, the holostean *L. oculatus* and the chondrostean *A. ruthenus*, stretching in the latter into more superficial layers of the optic tectum, but leaving the stratum opticum devoid of labeled cells (Fig. 7a–d). In addition, the optic tectum of the teleost *D. rerio* housed scattered SATB1/2-ir cells in the periventricular layer (Fig. 7e, f). Apart from the optic tectum, the torus semicircularis and the mesencephalic tegmentum also presented populations of cells labeled for SATB1 in the non-teleost actinopterygian species (Fig. 7a–c), while these areas showed SATB1/2 immunoreactivity in the lungfish (Fig. 8a). In the case of *D. rerio*, only the mesencephalic tegmentum was labeled with SATB1/2 (Fig. 7e).

In the rostral rhombencephalon, SATB1 labeling was detected in cells of the central gray of all fishes studied (Fig. 8c), with the exceptions of *D. rerio* and *P. dolloi*, whose central gray presented cells labeled for SATB1/2 (Fig. 8b, d). Strikingly, SATB1/2-ir cells were observed in the interpeduncular nucleus of the cladistians species, ventrally to the superior raphe nucleus (Fig. 9e), while the holosteans *L. oculatus* showed labeled cells in the ventral octavolateral area (Fig. 9f). The reticular formation showed in its superior and median portions SATB1 immunoreactivity in cladistians and *A. ruthenus* (Fig. 9h, i), and SATB1/2 in *L. oculatus* and the lungfish (Figs. 8c and 9a, c, g). Only in the case of the lungfish the inferior portion also presented labeled cells (Fig. 9c), while cladistians were the only species to colocalize SATB1 and OTP in cells of the reticular formation, specifically in its median portion (Fig. 9h). In mid to caudal alar rhombencephalic levels, a remarkable population exclusively of SATB1-ir cells was observed in the facial lobe and solitary tract nucleus of non-teleost actinopterygian fishes, distinct of the TH-ir cells housed in this nucleus (Fig. 9d). Finally, no immunolabeling was observed in the spinal cord of any species studied.

## Discussion

### Protein sequence analysis and technical considerations

Comparing the SATB1 sequences in all fish models analyzed we found that, in general terms, it is very conserved in all of them, and in particular the functional domains (see Fig. 2). For the SATB2 sequence, it showed a lower degree of similitude compared to SATB1, especially in the case of teleosts and holosteans (see Fig. 3). However, the degree of similarity and therefore the conservation of the sequence is high.

A previous study about the SATB2 role in the craniofacial development provided a detailed insight into the conserved protein sequence and the expression pattern in mouse, chick and zebrafish (Sheehan-Rooney et al. 2010). The characterization of the full-length *SATB2* transcript in mouse, chick and zebrafish compared to human, indicated, as in our present results, that the predicted protein sequence of SATB2 is conserved, in particular, the SATB, CUT and homeobox domains (Sheehan-Rooney et al. 2010), suggesting that the functional implication is also conserved. The expression analysis showed that the spatial and temporal pattern of SATB2 in the species analyzed correlates with those structures affected in human SATB2 mutated patients (Britanova et al. 2006; Dobreva et al. 2006; see discussion in Sheehan-Rooney et al. 2010). In addition, after Satb2 morpholino injections in zebrafish, migration defects during gastrulation have been described at early developmental stages (Ahn et al. 2010), although later developmental alterations were not described, most likely due to unviability of the zebrafish embryos.

In the analysis of epitope sequences performed in the present study, the SATB1 antibody was designed against an amino acids sequence from the center of the SATB1 protein of human (see Fig. 2). When we analyzed the sequence in all models, including human, we observed that this antibody recognizes SATB1 with high specificity in the models analyzed, but not SATB2. Therefore, we conclude that the specificity we observed in the results is very reliable. In the case of zebrafish, we believe that the fail in the immunofluorescence experiments is due to an effect of protein folding in this teleost model as opposed to the rest of the fishes analyzed, since based on the sequence analysis we know that SATB1 is present (the gen is annotated in the forms a and b: ZFIN:ZDB-GENE-031010-36 and ZFIN:ZDB-GENE-030131-7105 respectively) and it shows a high degree of similarity in the epitope recognized by the antibody (present results).

The SATB1/2 antibody under analysis was designed against a recombinant fragment corresponding to human SATB2 in the C-terminal end of the protein (see Table1 and Fig. 3). In particular, this antibody recognizes amino acids starting at 601 in the C-terminus of the human SATB2 protein (Uniprot: Q9UPW6). According to our present results, this region of the protein is very similar, in the species tested including human, to that present in the SATB1 protein (see Table 2). In addition, it was recently indicated by the commercial supplier in the specifications of the product that this antibody targets SATB1 and SATB2 proteins. According to our results, it is possible that this antibody recognizes both proteins in all species studied, although SATB2 with a higher degree of specificity.

The present protein analyses confirmed the high degree of conservation of both SATB1 and SATB2 proteins, in particular their functional domains (see color codes in Figs. 2 and 3), and therefore, the reliability of the results obtained by immunohistochemistry is very high, but we cannot exclude that we are detecting both populations with the SATB1/2 antibody.

### The neuroanatomical expression pattern

In the present comparative study, we have analyzed the immunolabeling pattern of SATB1 and SATB2 in the brain of key fishes, using in all cases the same commercial antibodies under the same technical conditions (see “Materials and methods” section).

SATB1 and SATB2 expression patterns in the brain have been described in detail in mouse (Huang et al. 2011, 2013), but in these studies, based on the antibodies used for their analyses, we consider that the description of SATB2 may correspond to both proteins. This is because many of the currently commercially available antibodies against SABT2 are designed (like the *abcam* antibody used in the present study) against a region of the protein very similar to that of SATB1. We will discuss each case in detail. In other amniotes, SATB2 expression has been correctly described in the pallium of chicken (García-Moreno et al. 2018), turtle (Suzuki and Hirata 2014), and alligator (Briscoe and Ragsdale 2018), but detailed data are lacking in the rest of the brain, as well as in anamniotes, except for zebrafish (Ahn et al. 2010; Pradhan et al. 2021). Moreover, it is particularly striking that there are no data on SATB1 expression in non-mammalian vertebrates. Thus, in the present study we conducted a comprehensive analysis of SATB1 and SATB2 immunolocalization in the brain of key osteichthyanfishes, selected because of their special evolutionary relationships (see Fig. 1A). In particular, we have generated a genoarchitectonic map of evolutionary SATB1 and SATB2 expressions that is consistent with the prosomeric model, but with notable differences in expression patterns between sarcopterygians and actinopterygians fishes that point to important divergent features in these vertebrate brain areas (Fig. 10). In the following sections, we will discuss our results in the context of existing data in amniotes and zebrafish for each main region of the brain.

#### Forebrain

**Olfactory bulb and pallium.** In the olfactory bulbs of mice it has been described *Satb1* gene expression in both neuronal and non-neuronal cell populations (Chang and Parrilla 2016). However, at protein expression terms, in adult mouse no SATB1 positive neurons were described in the main and accessory olfactory bulbs (Huang et al. 2011). In our analysis we have detected the presence of a population of SATB1 cells in the olfactory bulb of actinopterygian fish, contrary to what has been observed in sarcopterygians, both in lungfish (present results) and in other tetrapod models (such as amphibians or reptiles; data not shown). This data is very interesting as it results in a discrepancy at this evolutionary point, but it also raises the need to analyze in other models of sarcopterygians what is happening during development, since it could be similar to mammals, in which its expression is lost in the adult stage, or the contrary, with this ancestral characteristic of actinopterygian fish lost in these non-mammalian models.

In the case of Satb1 in the adult mouse cortex, positive neurons were abundant in the deep layers of the neocortex, subiculum, and anterior olfactory nucleus (Huang et al. 2011). Satb2 has been shown to be expressed with other cell identity transcription factors (CITF genes) in birds, reptiles, and mammals (Nomura et al. 2018; Tosches et al. 2018; Tosches and Laurent 2019). In mammals, Satb2 was abundantly expressed in all six layers of the neocortex (Britanova et al. 2005, 2008; Alcamo et al. 2008; Nielsen et al. 2010; Balamotis et al. 2012; Zhang et al. 2012; Huang et al. 2013; Martins et al. 2021), especially in the intracortical projection cells through the corpus callosum (Paolino et al. 2018), repressing the extracortical projection neuron identity in these cells.

The present results in the sarcopterygian and actinopterygian models analyzed allow us to conclude that the extensive expression of SATB1 and SATB2 in the pallium is an acquired characteristic of sarcopterygians, since only in the case of lungfish we found a discrete expression in the pallial region, which is totally absent in the case of actinopterygians. The absence of SATB1 and SATB2 in the pallium of the actinopterygian fishes suggests a null participation of these transcription factors in the mechanisms implicated in the pallial formation, although additional data in development are necessary to confirm this idea. In evolutionary terms, it makes sense, since Satb2 has been implicated in the evolution of the neocortex through different mechanisms, but in particular it has been shown that during cortical neurogenesis the control of Satb2 expression timing is crucial. For example, in higher primates, the expansion of supragranular cell layers has been linked to the delayed appearance of the Satb2 protein (Otani et al. 2016; Paolino et al. 2020; Martins et al. 2021). Satb2 may also relate to additional evolutionary acquisitions, as it is the case for callosal projecting cells (Paolino et al. 2018). The earliest indication of a distinct pallial commissure in vertebrates comes from the African spotted lungfish, a basal member of the lobe-finned fish lineage (Northcutt and Westhoff 2011; Suárez et al. 2014). This commissural tract has been considered homologous among tetrapods (called the hippocampal commissure in mammals), whereas the corpus callosum is unique to placental mammals (Suárez et al. 2014, 2018). In this context, lungfish is the first model in evolution with pallial expression of SATB1- and SATB2-ir cells and a pallial commissure, in contrast to actinopterygians (present results). This is a very interesting difference, since its probable functional implication in the pallium evidences the key involvement of this gene in cortical evolution and opens an important line in the evolutionary analysis of the pallium.

**Subpallium.** In all models analyzed in the present study, scattered SATB1- and SATB2-ir cells have been observed in several subpallial structures (see Table 3 and Fig. 10). In the mouse subpallium, SATB1 has been reported in many positive neurons in the medial septal nucleus, the nucleus of the diagonal band, the bed nucleus of the stria terminalis (BST) and the substantia innominata (Huang et al. 2011). Moreover, SATB2 has been described in scattered cells in the BST and the diagonal band (Huang et al. 2013). As indicated above, it is possible that in these studies SATB2 expression also shows SATB1 expression due to the non-specificity of the antibody used. In the lungfish, we observed SATB1- and SATB2-ir cells in the BST and in the septum. In the case of actinopterygians, SATB1 and SATB2 expressing cells were detected in Vv, a region that may be genoarchitectonically and neurochemically interpreted as homologous of the septum and the BST (Moreno et al. 2012; González et al. 2014; López et al. 2016). Therefore, it seems to be a primitive and conserved feature.

Additionally, during development in mouse, it has been described that Satb1 is a key regulator of cortical interneuron development, required for the maturation of medial ganglionic eminencie (MGE)-derived cortical interneurons. In particular, Satb1 functions downstream of Lhx6 to control the transition of immature migrating interneurons into the differentiated somatostatin subtype (Denaxa et al. 2012). Moreover, conditional removal of Satb1 in mouse interneurons results in the loss of a majority of somatostatin-expressing cells across all cortical layers, as well as some parvalbumin-expressing cells in layers IV and VI, by postnatal day 21 (Close et al. 2012). We do not have data on SATB1 expression during development in the models analyzed, thus we do not know the expression in the MGE, but in adult, we did not find expression in pallial interneurons, in contrast to those observed in non-mammalian tetrapods (own unpublished data). However, the presence of somatostatin and parvalbumin-positive interneurons have been described in the pallium of *Polypterus senegalus* (Jiménez et al. 2020), therefore, although we have not detected SATB1 and SATB2 expression in the pallium of these adult models, we cannot rule out their involvement during development in the specification of these populations.

In the case of the amygdala, in mouse SATB1-positive neurons were described in the anterior part of the basolateral amygdaloid nucleus and, to a lesser extent, in the medial amygdaloid nucleus (Huang et al. 2011). Few SATB1- and SATB2-ir cells were observed in the caudal part of Vd of cladistians, chondrosteans and teleosts, a region that might correspond to the central amygdala of lungfishes and amphibians, based on the presence of nitrergic cells, among other features (Moreno and González 2005; González and Northcutt 2009; López et al. 2016). Still regarding the amygdaloid complex, scattered SATB1- and SATB2-ir cells have been detected in the medial amygdala of lungfish, as well as in the actinopterygian Vs and Vp regions (present results), suggesting that it seems highly conserved. The region identified in the caudal telencephalon of ray-finned fishes as Vs and Vp could be the actinopterygian representative of the medial amygdala of tetrapods, attending to the OTP expression and other neurochemical features (Moreno and González 2003; González et al. 2010; 2014; López et al. 2016, 2022), as it probably corresponds to the posterior and dorsal portions of the medial amygdala of zebrafish (Porter and Mueller 2020).

**Preoptic area.** The high presence of SATB1- and SATB 2-ir cells found in the preoptic region is probably the most conserved feature in all vertebrates analyzed in the present study. The regionalization of this territory is evident by the combination with TH, expressed in an extensive population in this region, although generally no double-labeled cells were found in any model. In addition, combined analysis with OTP, one of the main markers of the paraventricular hypothalamic region, revealed the boundary between both regions. Furthermore, SATB1 and SATB 2 expression was found in the acroterminal region of the preoptic area, including interestingly a significant population of double-labeled TH cells. This population is attributed to the acroterminal domain (Puelles and Rubenstein 2015), and it is important to note that this area is a direct derivative of (or occupies territory previously occupied by) the alar acroterminal organizer. Similarly, in mammals Satb2 has been described in basal regions in derivatives of the acroterminal domain, also derived from the acroterminal basal organizer (see below; Ferran et al. 2015), which suggests the likely important involvement in the specification of this domain. However, no SATB1 expression has been described in mouse (Huang et al. 2011).

**Hypothalamus.** In adult mice, SATB1 labeled neurons were observed in the medial tuberal nucleus, posterior hypothalamic area, ventral part of the premamillary nucleus and supramamillary nucleus (Huang et al. 2011), whereas SATB2 expression has been reported in the lateral hypothalamic area, the arcuate nucleus, the paraventricular nucleus (Huang et al. 2013) and the ventromedial nucleus (Glendining et al. 2020). Furthermore, in a recent re-evaluation of the hypothalamic organization it has been described that Satb2 expression during mouse development is restricted to the basal portion of the terminal hypothalamic prosomere, especially in the anterobasal nucleus, the tuberal domain and the ventromedial nucleus (Ferran et al. 2015). In our analysis, in the alar hypothalamus we detected cells very discretely in the paraventricular region of all models, probably migrated from the adjacent preoptic area. All models analyzed expressed SATB2-ir cells in the subparaventricular area, in particular in the suprachiasmatic nucleus (SC), but no double-labeled cells were observed within the TH population, with the exception observed in the lateral part of the SC of zebrafish. Thus, it seems to be a primitive and conserved feature, lost in mammals, and suggests the importance of this family of homeodomain proteins in the specification of this structure, or in any of its populations.

In the mouse basal hypothalamus, it has been described that, along with its involvement in the regionalization of the hypothalamus (Britanova et al. 2005; Kurrasch et al. 2007), Satb2 is important in the establishment of hypothalamic circuitry at specific times in development, in particular in the establishment of normal projections from the arcuate nucleus (Bouret and Simerly 2006). Furthermore, SATB2 is specifically expressed in the A12 group of hypothalamic dopaminergic neurons (Huang et al. 2013) and in the ventromedial hypothalamus (Kurrasch et al. 2007). In Satb2 KO mice, only the dopaminergic neurons in the arcuate nucleus were affected, due to a defective postmitotic differentiation in the absence of Satb2 (Zhang et al. 2021), revealing the involvement of Satb2 in the development of this population. Additionally, the arcuate nucleus characteristically expresses the transcription factor OTP (Acampora et al. 1999; Bardet et al. 2007), which is involved in somatostatin phenotype specification (Kurrasch et al. 2007; Lee et al. 2018). What we observed in the tuberal hypothalamic region, equivalent to the arcuate nucleus, is that SATB1- and SATB2-ir cells coexpress OTP, but not TH in lungfishes (present results), although it does in mammals (Zhang et al. 2021). Therefore, this region shows a peculiar expression that is likely related to the functional implication of these proteins in the specification of particular populations in this area. In our analysis we have detected the presence of a population of SATB1 cells in the mamillary region of the most basal models of actinopterygian fish, comparable to that described in mammals in the premamillary and supramamillary nucleus (Huang et al. 2011). Once again, in this case we are faced with the possibility of an ancestral feature missing in the rest of the scale, or a feature related to the adaptability of cell types. In this line, the hypothalamus of actinopterygian fishes shows many conserved and divergent structures and characteristics among vertebrates, like the inferior hypothalamic lobe and the saccus vasculosus, a peculiar sensor of seasonal changes in day length. Therefore, in this case, the evolutive acquisition, given the lack of expression of SATB1- and SATB2-ir cells in the basal hypothalamus of actinopterygian fishes, supports its importance in the specification of this region, and/or its connectivity in sarcopterygians. Moreover, this situation of both conserved and acquired characteristics makes this region an attractive subject for evolutionary studies.

**Diencephalon.** Within the diencephalon, only two thalamic nuclei have been described containing SATB1-positive neurons in mice, the centrolateral thalamic nucleus and the parafascicular thalamic nucleus (Huang et al. 2011). Among the models analyzed, only lungfish showed SATB1- and SATB2-ir cells in the thalamus, specifically, in scattered cells in the anterior and central nucleus. It seems to be an acquired feature by sarcopterygians, since the analyzed actinopterygian models lack SATB2-ir cells in this prosomere, but not SATB1 (see Table 3). This may be very interesting to analyze in terms of the implication of Satb2 in this functional circuit, since sarcopterygians are the only ones with pallial SATB1- and SATB2-ir cells, as opposed to actinopterygians, thus constituting an evolutionary novelty. The hodological relationships between the thalamus and the pallium, minor in the case of anamniotes, but more extensive in amniotes, could be related to this differential expression. Similarly, a novel evolutive acquisition observed exclusively in the brain of sarcopterygians, and so absent in all actinopterygians studied, is the presence of SATB2-ir cells in the prethalamus, in contrast to the actinopterygians, which only showed SATB1 (present results). From the point of view of cellular evolution and functional implications of these genes, this is very interesting, since it may be the case that SATB1 in these models is supplying the functions of both genes or that it is a necessity/acquisition at this moment of evolutionary transition.

#### Brainstem

In the mouse brainstem, the vast majority of the SATB1 cells were dopaminergic. In particular, SATB1-positive neurons were observed in the superior colliculus, the tegmental nuclei, the parabrachial nucleus, the substantia nigra pars compacta, the ventral tegmental area, the retrorubral field, the nucleus of the trapezoid body, the periolivary region, and the nucleus of the lateral lemniscus. In addition, the presence of SATB2 has also been reported in the ventral tegmental area, the laterodorsal tegmental nucleus, the dorsal raphe nucleus, the rostral periolivary region, and the parabrachial nucleus. It is also specifically expressed in serotonergic neurons in the dorsal part of the dorsal raphe nucleus (Huang et al. 2013). Therefore, there are some areas of coexpression of both proteins, although the SATB2 antibody could be detecting only SATB1 due to its specificity, as detailed in previous regions.

Within the midbrain of the analyzed models, only in sarcopterygians we have found SATB1- and SATB2-ir cells in the torus semicircularis and mesencephalic tegmentum, but SATB1 is expressed in both regions of all models. In the rhombencephalon, only the cladistian fishes show SATB2-ir cells in the raphe area, but no coexpression with serotonin was observed (present results). Although the dorsal raphe neurons have been described to express SATB2 in mouse (Huang et al. 2013), recent analyses by single-cell RNA sequencing and systematic in situ hybridization of the dorsal and medial raphe nuclei mapped specific clusters (differentially expressing neuropeptides, receptors, ion channels, and transcription factors), which did not include Satb2 (Ren et al. 2019). What has been described in postnatal mice, however, is that in the absence of 5-HT transporters, serotonergic innervation increased in the postnatal prefrontal cortex, but the Satb2-positive intracortical projection neurons decreased (Witteveen et al. 2013). Therefore, the possible presence of Satb2 in serotonergic cells of the raphe nuclei seems to be a novel and specific feature acquired in mammals.

Finally, in mammals, the parabrachial nucleus (Pb) is a mixed population, composed of different subpopulations, including Satb2-expressing neurons (Karthik et al. 2022). These subpopulations are hodologically related with many brain regions like the amygdala, the hypothalamus, and the hindbrain, participating in many different homeostatic functions. In addition, the Satb2 glutamatergic neurons of the Pb project to the cortical territories (Grady et al. 2020), in particular to regions associated with taste, including the gustatory thalamus and insular cortex (Jarvie et al. 2021). It is also interesting to note that projections from the insular cortex and the claustrum to subregions of the Pb containing cortical projection neurons have been described in mammals (Grady et al. 2020). Specifically, it has been observed that Satb2 neurons in the Pb respond to all five basic taste modalities (Jarvie et al. 2021), playing a critical role in sweet taste transduction (Fu et al. 2019). The sense of taste plays a key role in animal survival through an animal's reflex decision to swallow or reject a potential food item. In fish, taste information is mainly located in the secondary gustatory nucleus, originating in the isthmic region (Finger 2009). The absence of SATB1 and SATB2 cells in this nucleus in fish suggests that this acquisition appears later in evolution. However, given its functional importance and the degree of conservation in tetrapods (its existence has been demonstrated in amphibians and reptiles; data not shown), it is possible that some of the described populations lodged along the anterior reticular chain proximal to this region are involved in this functionality. Further experiments in this direction would be interesting.

## Concluding remarks

We have analyzed the SATB1 and SATB2 protein sequences and their brain expression patterns in specific bony fish models at the base of gnathostomes. The first evidence shown is the high degree of conservation of these proteins in the base of evolution, in general in almost all the sequence, but especially in the functional domains, indicating that most probably the functional implications of these proteins are of great importance for survival, as demonstrated by their conservation in evolution. In this sense, these functional implications must be especially related to early developmental processes, as it has been described in mammals, since, as we can see in the analysis of the expression of both proteins in the brain, significant evolutionary divergences are observed between actinopterygians and sarcopterygians, even with the high degree of conservation they present. A striking example of this is the pallial region, where most probably both SATB1 and SATB2 are involved in the evolutionary process of this region, and more specifically in cortical expansion and cell type specification, due to the lack of expression in actinopterygian models, all of them with a pallium developed by a process of evagination.

Finally, in this sense, in the case of SATB2, alterations in this gene are related with the SATB2-associated syndrome, which causes craniofacial anomalies, among other pathologies (reviewed in Zarate et al. 2017). Similarly, the involvement of Satb2 in craniofacial development has been described in other models (Sheehan-Rooney et al. 2010). This is why it would be of great interest to study the expression of this gene and the functionality of this protein in agnates, in order to know its functional implication in these jawless vertebrate models.

## Data Availability

The raw data that support the findings of this study are available on request from the corresponding author.

## Abbreviations

ac: Anterior commissure
A.r: *Acipenser ruthenus*
Amy: Amygdaloid complex
BST: Bed nucleus of the stria terminalis
Cb: Cerebellum
cCb: Crista cerebellaris
Cg: Central gray
chp: Choroid plexuses
D.r: *Danio rerio*
Dm: Medial zone of the dorsal telencephalic area
DP: Dorsal pallium
E.c: *Erpetoichthys calabaricus*
fb: Forebrain bundle
flm: Medial longitudinal fascicle
fr: Fasciculus retroflexus
gl: Glomerular layer of the olfactory bulb
igl: Internal granular layer of the olfactory bulb
Ip: Interpeduncular nucleus
Ipn: Interpeduncular neuropil
L.o: *Lepisosteus oculatus*
Lc: Locus coeruleus
LP: Lateral pallium
Ma: Mamillary hypothalamic area
MeA: Medial amygdala
MP: Medial pallium
Nsol: Nucleus of the solitary tract
ob: Olfactory bulb
oc: Optic chiasm
OT: Optic tectum
P.d: *Protopterus dolloi*
P.s: *Polypterus senegalus*
p1–p3: Diencephalic prosomeres 1–3
pA: Pallial amygdala
Pa: Paraventricular hypothalamic area
P.d: *Protopterus dolloi*
POA: Preoptic region
POAr: Rostral preoptic area
PT: Pretectum
PTh: Prethalamus
r0: Rhombomere 0
r1–8: Rhombomeres 1–8
Ras: Superior raphe nucleus
Ri: Inferior reticular nucleus
Rm: Median reticular nucleus
Rs: Superior reticular nucleus
RTu: Retrotuberal hypothalamic area
S: Septum
sco: Subcommissural organ
sfgs: Stratum fibrosum et griseum superficiale of the optic tectum
sgc: Stratum griseum centrale of the optic tectum
SN: Substantia nigra
sol: Solitary tract
SP: Subpallium
SPa: Subparaventricular hypothalamic area
SPar: Rostral subparaventricular hypothalamic area
spv: Stratum periventriculare of the optic tectum
Str: Striatum
Tegm: Mesencephalic tegmentum
Th: Thalamus
Tl: Torus longitudinalis
TP: Posterior tubercle
Ts: Torus semicircularis
Tu: Tuberal hypothalamic area
v: Ventricle
vCb: Cerebellar valvula
Vd: Dorsal nucleus of the ventral telencephalic area
Vl: Lateral part of the ventral telencephalic area
Vp: Posterior nucleus of the ventral telencephalic area
VP: Ventral pallium
Vs: Supracommissural nucleus of the ventral telencephalic area
Vv: Ventral nucleus of the ventral telencephalic area
Zip: Periventricular nucleus of the zona incerta
VIIIv: Ventral octavolateral area
